# The Microscope

**Published:** 1877-11

**Authors:** 


*Practical Hints on the Selection and Use of the Microscope. Intended for beginners.* By JOHN PHIN, Editor of "The American Journal of Microscopy." New York: The Industial Publication Company. 1877. Price, 75 cts.

The use of the microscope has become so world-wide that a knowledge of it is a necessity to a liberal education. Although the eye may be accostomed to its use there are many questions in relation to objects, testing objects, and mounting specimens with which one may be unacquainted. This small volume is to supply the want.

The author does not make an opportunity to write a text book upon the general principles of botany, zoology or histology but confines himself to the object he declares on the title page and presents a guide to the best methods of using the microscope. It is intended for the beginner and contains all that is necessary to a thorough practical knowledge of the microscope and its uses. The chapters upon choosing a microscope and mounting objects will be found of the greatest value. The price is so low as to place it within the reach of everyone. C.

